# Case Report: Modified therapeutic strategy for corrosive esophageal stricture—endoscopic balloon dilation combined with acellular dermal matrix transplantation

**DOI:** 10.3389/fmed.2026.1849683

**Published:** 2026-06-24

**Authors:** Xinhui He, Li Wang, Zhao Mu, Shuang Liu, Yong Yan, Lin Zhang, Jiamin Qin, Liming Wen

**Affiliations:** 1 Department of Gastroenterology, The Affiliated Hospital of Southwest Medical University, Luzhou, Sichuan, China; 2 Department of Gastroenterology, Mianyang 404 Hospital, Mianyang, Sichuan, China

**Keywords:** acellular dermal matrix, case report, caustic ingestion, endoscopy, esophageal stricture, therapy

## Abstract

Esophageal stricture caused by corrosive substance ingestion typically presents with dysphagia, chest pain, vomiting, and other discomfort, which severely impairs patients’ quality of life. The treatment of corrosive esophageal stricture remains a challenging clinical issue that urgently needs to be addressed, and there have been no previous reports on the use of endoscopic balloon dilation combined with acellular dermal matrix transplantation for the treatment of corrosive esophageal stricture. Herein, we present a case of a 21-year-old male patient who developed severe panesophageal stricture following accidental ingestion of acidic toilet cleaner. Despite 13 prior endoscopic interventions, the patient suffered from persistent stricture recurrence and refractory symptoms. Subsequent treatment with combined endoscopic balloon dilation and acellular dermal matrix transplantation was performed successfully, leading to short-term significant symptomatic relief and improved quality of life. This case report demonstrates the feasibility and potential efficacy of the combined use of endoscopic balloon dilation and acellular dermal matrix transplantation for the management of severe panesophageal strictures induced by corrosive ingestion, providing a potential therapeutic option for this challenging clinical entity.

## 1 Introduction

Corrosive esophagitis, an inflammatory condition of the esophagus resulting from ingestion of caustic acidic or alkaline substances, may lead to serious complications such as esophageal cicatricial stricture (1, 2). Based on the Zargar classification, stricture occurs in 61.5–100% of patients with grade ≥2b mucosal injury, typically developing within 2 months after injury (3–6). Therapeutic approaches for corrosive esophageal stricture include endoscopic dilation, temporary stent placement, intralesional steroid or mitomycin, and surgical intervention (5). Endoscopic dilation is the primary treatment option, but multiple dilations are often required to achieve the desired effect (7). The resulting multiple hospitalizations increase the economic, physical, and psychological burden on patients. Surgical intervention can be an option for esophageal stricture, but careful case selection is necessary, and there are associated surgical risks. Therefore, the key issue is how to safely and effectively treat corrosive full-length esophageal stricture that cannot be surgically reconstructed, and the treatment plan is still under continuous exploration. we report a patient with esophageal stricture secondary to caustic ingestion who underwent combined endoscopic balloon dilation and acellular dermal matrix transplantation. Currently, research on the application of acellular dermal matrix in esophageal stricture focuses on prevention, mainly preventing esophageal stricture after endoscopic submucosal dissection. In this case, acellular dermal matrix transplantation is used as an adjuvant treatment for esophageal stricture caused by the ingestion of corrosive substances.

## 2 Case description

**Figure 1:**
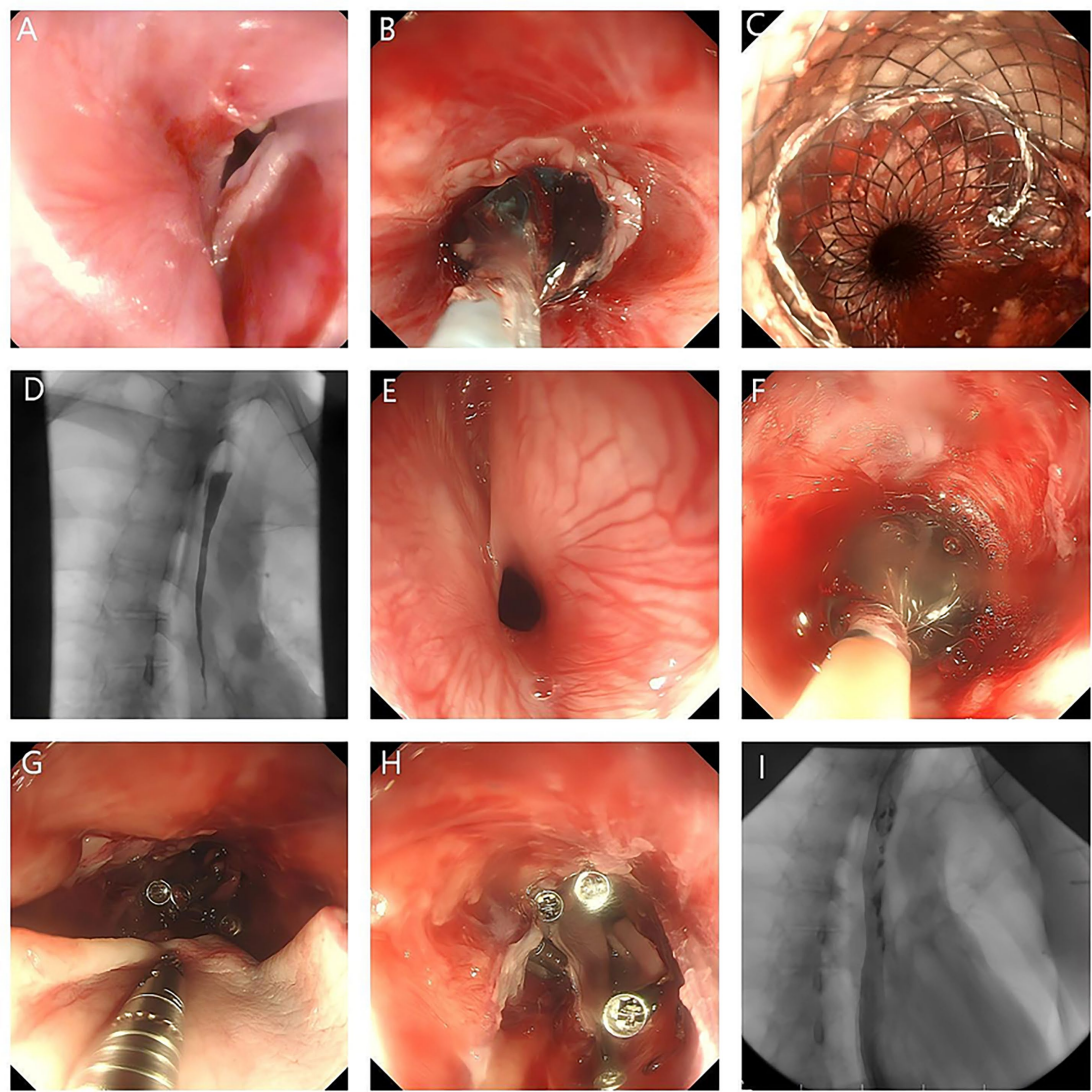
Imaging findings and endoscopic treatment in the patient. **(A)** Initial endoscopy revealed esophageal stricture. **(B)** Endoscopic balloon dilation was performed. **(C)** Fully covered esophageal stent placement was performed. **(D)** Esophagography demonstrated panesophageal stricture. **(E)** Endoscopy showed esophageal stricture at 17 cm from the incisors. **(F)** Endoscopic balloon dilation was performed. **(G,H)** Allogeneic dermal grafting was performed. **(I)** Postoperative esophagography revealed a patent esophageal lumen.

A 21-year-old male patient presented to our hospital with a 1-year history of dysphagia that had recurred 1 week prior. One year ago, the patient developed dysphagia, a sensation of obstruction when drinking water, hoarseness, and retrosternal pain after accidentally ingesting 40 mL of an acidic toilet cleaner containing surfactant, oxalic acid, fragrances, and preservatives. According to the Stooler’s Dysphagia Score (8), the dysphagia was graded as Stooler grade IV. The patient had been hospitalized and treated in several other hospitals successively. After relevant examinations, he was diagnosed with corrosive esophagitis and esophageal stricture. No significant improvement was observed after symptomatic treatment. Consequently, the patient presented to our hospital for further treatment. At the initial admission, the patient’s height was 183 cm, weight was 44 kg, and body mass index was 13.14 kg/m^2^. Given the patient’s severe malnutrition, young age, and extensive esophageal stricture, multiple hospitals declined surgical intervention. Furthermore, the patient and his family refused surgery and opted for endoscopic treatment instead. From the first treatment at our hospital to the present, the patient has undergone 13 endoscopic procedures (Table 1), including repeated balloon dilation, placement of temporary fully covered self-expanding metal stents and nickel-titanium memory alloy stents, and IT knife fan-shaped incision treatments (Figures 1A–C).

**Table 1:** Patient treatment process timeline.

Time	Therapeutic method	Balloon pressure	Stent implantation
2023.12.28	Endoscopic balloon dilation	2.5 kPa	
2024.01.11	Endoscopic balloon dilation + incision treatment + stent implantation treatment	2.0–3.0 kPa	One 12.0 * 2.0 cm fully covered self-expanding metal stent was implanted at 23 cm away from the incisors
2024.03.07	Endoscopic balloon dilation + stent implantation treatment	2.0–2.5 kPa	One 6.0 * 2.0 cm nickel-titanium memory alloy stent was implanted at 35 cm away from the incisors
2024.04.09	Endoscopic balloon dilation	2.5 kPa	
2024.05.16	Endoscopic balloon dilation	2.5–3.0 kPa	
2024.07.02	Endoscopic balloon dilation + stent implantation treatment	2.5–3.0 kPa	One 10.0 * 2.0 cm fully covered self-expanding metal stent was implanted at 33 cm away from the incisors
2024.08.08	Endoscopic balloon dilation therapy + stent removal surgery	2.5 kPa	remove three stents
2024.09.11	Endoscopic balloon dilation	2.5–3.0 kPa	
2024.10.10	Endoscopic balloon dilation + stent implantation treatment	2.5 kPa	One 12.0 * 2.0 cm fully covered self-expanding metal stent was implanted at 23 cm away from the incisors
2024.11.21	Endoscopic balloon dilation + stent implantation treatment	2.0–3.5 kPa	One 8.0 * 2.0 cm fully covered self-expanding metal stent was implanted at 19 cm away from the incisors
2025.02.18	Endoscopic balloon dilation	2.0–2.5 kPa	
2025.05.08	Endoscopic balloon dilation	2.0–2.5 kPa	
2025.06.24	Endoscopic balloon dilation + incision treatment	2.0–2.5 kPa	

Time is expressed as Year.Month.Day; Stent dimensions are shown as length × diameter; kPa, kilopascal; cm, centimetre.

At the time of this admission, the patient presented with dysphagia again. The patient could consume a liquid diet, but there was a feeling of choking when eating a semi-liquid diet, accompanied by acid reflux, heartburn, belching, and constipation. The Stooler grade of dysphagia was grade III. Upon this admission, his body mass index was 15.83 kg/m^2^, and the patient had moderate malnutrition. Other physical examinations were unremarkable. Laboratory findings were unremarkable. Esophagography showed stricture of the lumen in the upper and middle thoracic segments of the esophagus, with poor visualization of the lower esophagus, and the contrast medium did not reach the gastric cavity (Figure 1D). Based on the patient’s history, previous endoscopic findings, and imaging results, the final diagnosis was severe panesophageal cicatricial stricture and corrosive esophagitis.

An exploratory treatment of endoscopic balloon dilation combined with acellular dermal matrix transplantation was administered to the patient for severe panesophageal stricture. The procedure was as follows: After the patient was satisfactorily anesthetized with tracheal intubation, the endoscope was advanced to the esophagus. A stricture located 17 cm from the incisors had a luminal diameter of 0.5 cm (Figure 1E), while severe strictures at 26 cm and 34 cm from the incisors narrowed the lumen to 0.2–0.3 cm, which precluded endoscopic passage. Under direct vision, the Cook dilation balloon manufactured by Wilson - Cook Medical Incorporated was used for repeated dilation (Figure 1F). The balloon was inflated at a sustained pressure of 2.0–2.5 kPa for 30 s at a maximal dilation diameter of 1.8 cm. After that, the endoscope could pass through smoothly. During the operation, the entire esophagus was found to be stenotic. There was oozing of blood during the operation, and noradrenaline saline was sprayed for hemostasis.

A rotatable repetitive open-close soft tissue clamp (Micro-Tech (Nanjing) Co., Ltd.; model: ROCC - D - 26 - 195) was used to hold the acellular dermal matrix (3.0 cm × 4.0 cm; Beijing Jayyalife Biological Technology Co., Ltd). Under direct endoscopic visualization, we advanced the acellular dermal matrix into the esophagus and identified its adhesive surface. Twelve rotatable repetitive open-close soft tissue clamps were then applied to secure the acellular dermal matrix separately at the mid and lower esophagus (Figures 1G,H). The acellular dermal matrix was gradually and completely flattened to avoid wrinkles or poor fitting. Two pieces of acellular dermal matrix were used intraoperatively. They were transported at room temperature and stored at 4–8 °C.

Following endoscopic balloon dilation combined with acellular dermal matrix transplantation, the patient received intravenous cefuroxime sodium for infection prophylaxis, carbazochrome sodium sulfonate for hemostasis, and ilaprazole to prevent stress ulcer bleeding. Fasting, fluid replacement, and nutritional support were also provided. Esophagography performed on day 4 demonstrated patency of the esophageal lumen (Figure 1I), and chest CT revealed no contrast extravasation, esophageal rupture, perforation, or other complications. The patient was subsequently transitioned to a warm liquid diet. During the 4-month follow-up period, the dysphagia was graded as Stooler grade II. Four months later, the patient experienced recurrence of dysphagia accompanied by acid reflux, heartburn, belching, and constipation, with dysphagia progressing to Stooler grade III. Weight and body mass index were 53 kg and 15.83 kg/m^2^, respectively. Repeat gastroscopy revealed strictures in the upper and middle esophagus. The luminal diameter of the upper esophagus was approximately 0.5 cm, and that of the middle esophagus measured 0.8–0.9 cm. The lower esophagus remained patent. Endoscopic dilation treatment and stent placement treatment were provided.

## 3 Discussion

Strictures secondary to corrosive ingestion are mostly complex esophageal strictures, which are defined as lesions ≥2 cm in length, angular and irregular morphology, or severe luminal stenosis. Such strictures are difficult to treat and likely to develop into recurrent or refractory strictures (9). The patient in this case suffered from severe panesophageal stricture complicated by marked fibrosis and tissue stiffness. After repeated endoscopic balloon dilation and IT knife fan-shaped incision, the endoscope still could not pass through some parts of the esophagus. Stent placement over a metal guidewire was required to reestablish luminal patency.

Surgical interventions typically involved esophageal replacement with either a gastric or colonic pull-up (7). Although surgery provides long-term symptomatic improvement in carefully selected cases, it carries higher risks of complications and mortality compared to endoscopic therapy. Surgical complications may include aspiration pneumonia, injury to adjacent critical structures, and anastomotic strictures (10, 11). In the present case, the patient’s young age, severe malnutrition, and severe cicatricial stricture of the entire esophagus rendered surgical intervention highly challenging. As the patient declined surgery, endoscopic treatment was continued, with ongoing exploration of optimized therapeutic strategies.

Endoscopic dilation is the first-line treatment for esophageal strictures. It relieves dysphagia promptly but carries risks of bleeding, pain, and potentially life-threatening perforation (2, 12). To minimize patient discomfort associated with repeated dilation sessions, reduce complication rates, enhance therapeutic efficacy, lower stricture recurrence, and prolong symptom-free intervals, endoscopic dilatation is often employed in conjunction with adjunctive therapies. Common adjuvant approaches include intralesional steroid or mitomycin injections, endoscopic incision therapy, and stent placement (9). However, studies indicate that for corrosive-induced esophageal strictures, local steroid injections do not significantly reduce the frequency of dilation or prolong the symptom-free interval (13). Topical application of mitomycin represents another strategy to enhance the efficacy of endoscopic dilation, with some studies confirming its benefits in corrosive strictures (14). Nevertheless, standardized protocols for this approach remain lacking, and more large-scale, high-quality studies are needed to determine the optimal dosage, mode of administration, and patient selection for mitomycin C in refractory esophageal stricture (9). In this case, instead of using intralesional steroid or mitomycin C injections alongside endoscopic dilation, we made exploratory adaptation to the adjuvant therapy and adopted endoscopic dilation combined with acellular dermal matrix transplantation.

Regenerative medicine and tissue engineering have emerged as prominent research directions in the prevention and treatment strategies for benign esophageal strictures in recent years. Current approaches include autologous transplantation, cell sheet transplantation, endoscopic injection of autologous cell suspensions, and extracellular matrix biomaterial transplantation (15–18). Acellular dermal matrix, which removes the cellular components while retaining elastin, keratan sulfate, laminin and collagen, has very low immune activity and will not induce any rejection. It provides a structural scaffold for soft tissue regeneration and revascularization through their extracellular matrix mesh for host cell integration and by their ability to modulate the wound healing response via intrinsic growth factors and extracellular matrix components (19, 20). Several studies have applied acellular dermal matrix to prevent esophageal stricture following endoscopic submucosal dissection. In our prior clinical practice, acellular dermal matrix was utilized in patients undergoing circumferential esophageal endoscopic submucosal dissection to prevent stricture formation, with follow-up endoscopy at 6 months confirming favorable wound healing without esophageal narrowing. Although the capacity of acellular dermal matrix to reverse fibrosis remains to be fully elucidated, it can serve as a biomaterial scaffold that facilitates tissue regeneration. Acellular dermal matrix becomes incorporated into the host tissue and is gradually replaced by native collagen, thereby promoting and sustaining the healing process while minimizing scar tissue formation (21). Thus, we hypothesize that acellular dermal matrix may attenuate esophageal fibrosis, subsequently slowing luminal narrowing and prolonging symptom remission. Current adjuvant therapies for esophageal stricture have certain limitations. Steroid injections have not demonstrated significant reduction in dilation frequency or extension of symptom remission (13), while optimal dosing, administration route, and patient selection criteria for mitomycin C remain undefined (9). In this case, the patient developed recurrent esophageal strictures at intervals of 1–2 months following stent placement. To date, no studies have applied allogeneic dermal matrix as an adjuvant therapy for esophageal stricture caused by corrosive ingestion. Accordingly, we carried out an exploratory modification of the treatment regimen and adopted acellular dermal matrix for corrosive esophageal stricture, with the aim of extending the remission period. The recurrent stricture observed in the mid esophagus and upper esophagus after 4 months may be attributed to severe muscular layer injury, fibroblast hyperproliferation and excessive collagen deposition at sites adjacent to the transplantation, or inadequate contact between the acellular dermal matrix and esophageal wall due to clip-based fixation. Notably, the lower esophageal lumen remained patent during follow-up, suggesting a potential therapeutic effect of the acellular dermal matrix transplantation in mitigating stricture progression in this segment.

This approach has certain limitations. Firstly, although favorable short-term outcomes were achieved, the patient developed dysphagia again 4 months after treatment. This indicates that endoscopic balloon dilation combined with acellular dermal matrix transplantation cannot fundamentally resolve corrosive esophageal stenosis, and recurrent stricture remains a concern. The long-term efficacy of this technique has not yet been fully elucidated. Further studies with longer follow-up periods and larger sample sizes are required to verify its long-term therapeutic effects. Secondly, the patient only presented for medical evaluation due to dysphagia and declined endoscopic or imaging examinations at scheduled follow-up visits. Therefore, standardized quantitative indicators reflecting dynamic changes in esophageal patency were not available. In future studies, we will implement a standardized regular follow-up protocol combined with serial quantitative imaging evaluations to generate more rigorous research findings. Thirdly, we noted a mild prolongation of symptom remission in the short term. Nevertheless, there is insufficient evidence to link this improvement to the regenerative function of acellular dermal matrix. Relevant experiments will be performed in subsequent studies to elucidate its exact role and molecular mechanisms.

## 4 Conclusion

Endoscopic balloon dilation combined with acellular dermal matrix transplantation yields favorable short-term outcomes in the treatment of esophageal stricture caused by corrosive substance ingestion, relieving clinical symptoms and improving patients’ quality of life. However, as this study represents a single exploratory case, multicenter prospective investigations are warranted to validate the efficacy and safety of this treatment modality for corrosive esophageal stricture. Furthermore, we propose that acellular dermal matrix transplantation administered after corrosive substance ingestion but prior to stricture formation may play a preventive role against esophageal stricture. Additional research is required to evaluate the efficacy and safety of this prophylactic approach.

## Data Availability

The original contributions presented in the study are included in the article/supplementary material, further inquiries can be directed to the corresponding authors.
